# High‐Pressure Stability and Electronic Properties of Sodium‐Rich Nitrides: Insights from First‐Principles Calculations

**DOI:** 10.1002/cphc.202401150

**Published:** 2025-03-06

**Authors:** Qiuyue Li, Qiuping Yang, Shuai Han, Fei Li, Yansun Yao, Guochun Yang

**Affiliations:** ^1^ State Key Laboratory of Metastable Materials Science & Technology and Hebei Key Laboratory of Microstructural Material Physics School of Science Yanshan University Qinhuangdao 066004 China; ^2^ Key Laboratory of Materials Modification by Laser Ministry of Education Ion and Electron Beams (Dalian University of Technology) Dalian 116024 China; ^3^ Department of Physics and Engineering Physics University of Saskatchewan Saskatoon, Saskatchewan S7 N 5E2 Canada

**Keywords:** sodium-rich nitride, phase transtion, electride, high pressure, first-principles calculations

## Abstract

Using first‐principles structure search calculations, we investigated the phase stability of sodium‐nitrogen (Na−N) compounds under high pressure. Our study reveals that increasing pressure promotes the formation of Na‐rich nitrides, leading to the prediction of three previously unreported stoichiometries: Na_2_N, Na_5_N, and Na_8_N. Notably, the electride Na_5_N undergoes a pressure‐induced structural transition from a *P*6/*mmm* to a *P*6_3_/*mmc* phase. This transformation is characterized by spatial reorientation and redistribution of interstitial anionic electrons (IAEs). In the *P*6_3_/*mmc* phase, IAEs adopt a zero‐dimensional, triangular‐like configuration, whereas in the low‐pressure *P*6/*mmm* phase, they form an interconnected, graphene‐like network. With increasing pressure, *P*6_3_/*mmc* phase undergoes a transition from metallic to semiconducting behavior due to the increased interaction between sodium and IAEs. Additionally, *C*2/*m* Na_8_N, featuring triangular‐ and ship‐like IAEs, is predicted to exhibit superconductivity. Our findings provide new insights into the behavior and stability of Na‐rich nitrides under high‐pressure conditions.

## Graphical abstract

This work highlights how pressure‐induced IAEs rearrangements influence the thermodynamic stability and electronic behavior of these compounds, including metal‐to‐semiconductor transitions in *P*6_3_/*mmc* Na_5_N and superconductivity in *C*2/*m* Na_8_N.

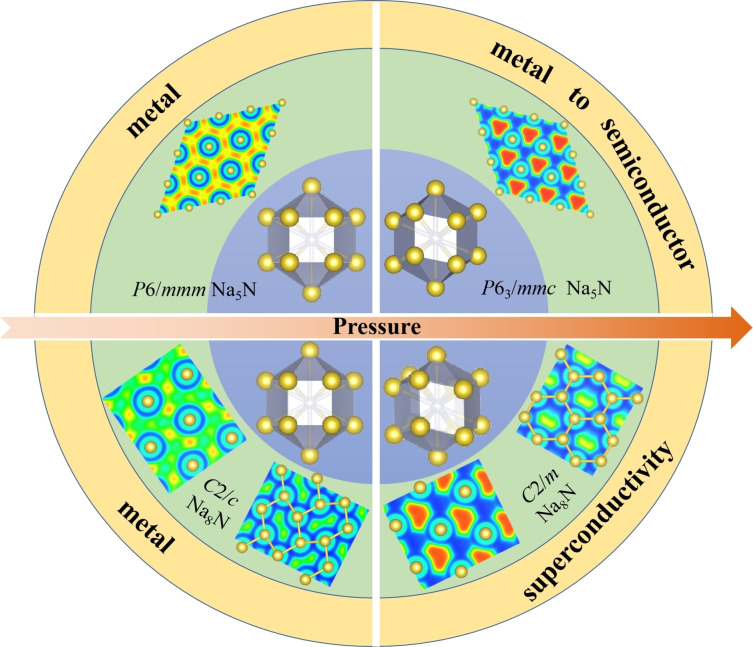

## 1 Introduction

Pressure, as a critical thermodynamic parameter, can significantly modulate the energy levels of atomic orbitals, bonding patterns, and material structures.[^1^, ^2^, ^3^] Consequently, it plays an irreplaceable role in fundamental scientific research and the development of new materials. Pressure has been shown to stabilize unconventional stoichiometric compounds, such as Na_3_Cl,^[4]^ Na_2_He,^[5]^ Fe_3_Xe,^[6]^ CsF_5_,^[7]^ and IrF_8_,^[8]^ challenging the classical understanding of chemical theories. Furthermore, it induces phenomena unobservable under ambient conditions, such as metal‐insulator transitions,^[9]^ and enables breakthroughs in superconducting transition temperatures,[^10^, ^11^] as exemplified by LaH_10_ achieving superconductivity above 250 K under high pressure.[^12^, ^13^] Pressure also facilitates the stabilization of a range of electrides with ultralow work functions and superconducting properties, paving the way for discovering and applying functional materials.[^14^, ^15^, ^16^, ^17^]

Alkali metals like lithium (Li) and sodium (Na), due to their unique valence electron configurations and diverse physicochemical properties, exhibit remarkable structural and properties under high pressure, attracting significant attention.[^9^, ^18^, ^19^, ^20^] In lithium systems, high‐pressure nitrides such as LiN_5_, theoretically predicted and experimentally synthesized, exhibit energy densities far exceeding conventional explosives like TNT and HMX.[^21^, ^22^] Li_5_N, predicted to have superionic characteristics under 150 GPa, demonstrates a superconducting transition temperature of 48.97 K.^[23]^ Similarly, high‐pressure sodium nitrides display diverse structures and novel properties. For example, NaN_3_ adopts three phases (*I*4/*mcm*, *P*6/*m*, and *C*2/*m*) under high pressure,^[24]^ and nitrogen‐rich compounds NaN_5_ and Na_2_N_5_ have been theoretically predicted and experimentally validated.[^25^, ^26^] Recent studies have identified four new nitrogen‐rich phases (*P*1 NaN_7_, *Cm* NaN_7_, *C*2 NaN_8_, and *R*‐3 NaN_8_) in the Na−N system at pressures of 50–100 GPa, with *Cm* NaN_7_ and *R*‐3 NaN_8_ showing potential applications due to their high energy density.^[27]^

Despite significant progress in nitrogen‐rich sodium compounds, the structures and properties of sodium‐rich compounds at higher pressures remain underexplored. Herein we systematically investigate the structures and properties of various Na_*x*_N_*y*_ (*x*=1–8, *y*=1–6) compounds at 0 K and pressures of 100, 200, and 300 GPa using first‐principles structure searching technology. Our findings reveal three new sodium‐rich stoichiometries: Na_2_N, Na_5_N, and Na_8_N. More interestingly, several novel structural units (e. g., Na_14_N double‐capped hexagonal prism, one‐dimensional Na chain, two‐dimensional Na network) are observed. Specifically, Na_5_N undergoes a phase transition from *P*6/*mmm* to *P*6_3_/*mmc* with compression, accompanied by spatial reorientation and redistribution of interstitial anionic electrons (IAEs). Notably, strong hybridization between IAEs and Na in *P*6_3_/*mmc*‐Na_5_N induces the electronic properties of transition from metallic to semiconducting, which contrasts with the *P*6/*mmm* phase with a single metallic property. These results provide new insights into the structural modulation and property tuning of sodium‐rich nitrides under high pressure.

## Computational Methods

We have carried out extensive structural searches calculations for the Na−N system using swarm‐intelligence‐based CALYPSO structure prediction method.[^28^, ^29^] Structural relaxation and electronic property calculations were performed using the Vienna *ab initio* simulation package (VASP),^[30]^ employing density functional theory (DFT).[^31^, ^32^] The Perdew‐Burke‐Ernzerhof (PBE)^[33]^ implementation of the generalized gradient approximation (GGA) was chosen for the exchange‐correlation functional.^[34]^ A Monkhorst‐Pack Scheme^[35]^ with a *k*‐point grid spacing of 2*π*×0.03 Å^−1^ and a kinetic energy cutoff of 750 eV were chosen to ensure good convergence of the total energy. Projector augmented wave (PAW)^[36]^ pseudopotentials, with valence electron configurations of 2*p* ^6^3 *s* ^1^ for Na and 2 *s* ^2^2*p* ^3^ for N, were used. The validity of adopted pseudopotentials under high pressure was confirmed with the full‐potential linearized augmented plane wave method, as implemented in the WIEN2k package^[37]^ (Supplemental Material Figure S1). To verify the dynamic stability of the Na−N compounds, phonon dispersion curves were performed using the PHONOPY code with the supercell finite displacement method.^[38]^ Superconducting properties were investigated within the framework of density functional perturbation theory, utilizing the QUANTUM ESPRESSO package.^[39]^ More computational details are provided in the Supplemental Material.

## 2 Results and Discussion

### 2.1 Phase Stability of Na−N Compounds

**Figure 1:**
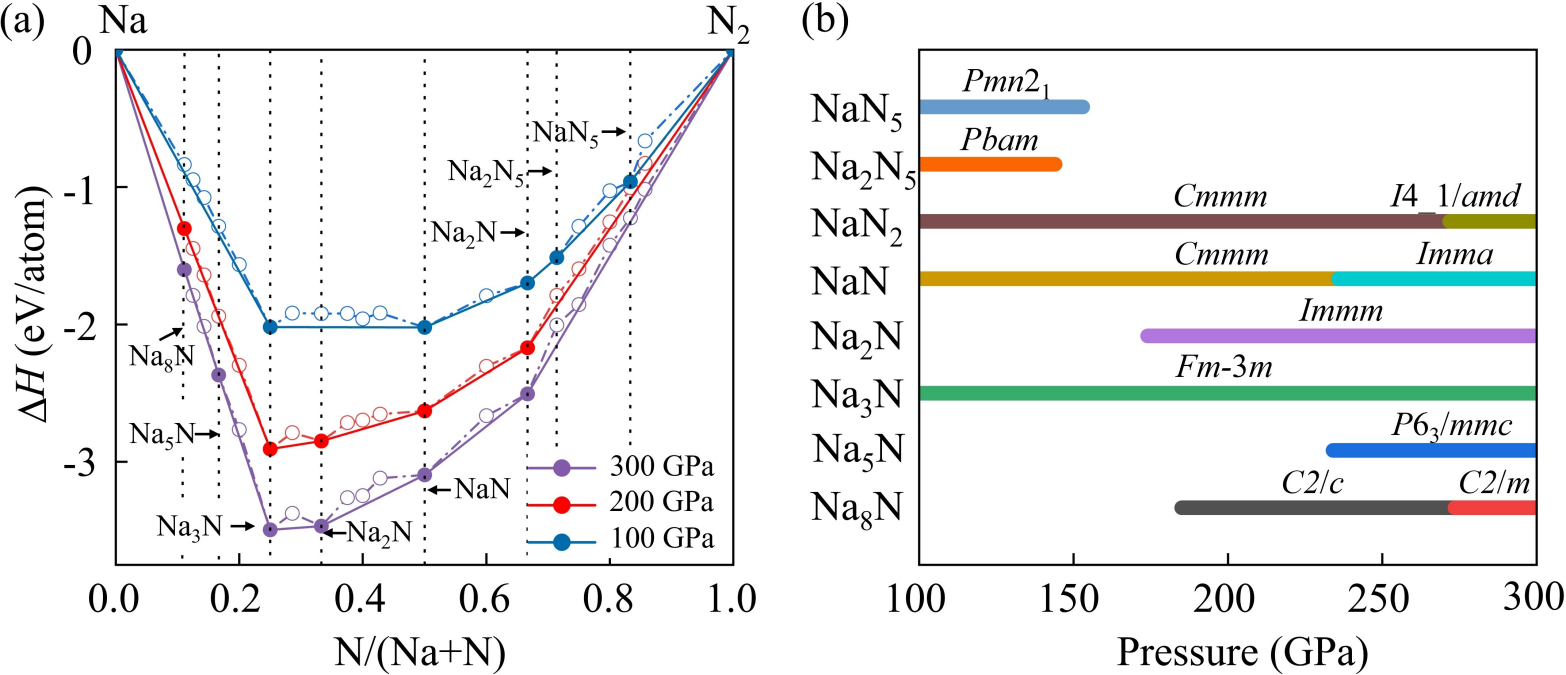
**(a)** Convex hull of the Na−N system under high pressure. (b) Pressure‐composition phase diagram of Na−N compounds.

To explore the phase stability of the Na−N system, we performed extensive structural searches on Na−N compounds with various Na_*x*_N_*y*_ (*x*=1–8, *y*=1–6) compositions at a temperature of 0 K and selected pressures of 100, 200, and 300 GPa. For each Na_*x*_N_*y*_ composition, the structure with the lowest energy was selected to evaluate the formation enthalpy relative to the elemental solids sodium[^19^, ^20^] and nitrogen.^[40]^ A convex‐hull diagram is then built by utilizing the formation enthalpies of the Na_*x*_N_*y*_ compounds (Figure 1a). In general, compounds that sit on the solid lines are thermodynamically stable, and can be synthesized in certain conditions. Nevertheless, those lying on the dotted lines are either metastable or unstable, depending on their kinetic energy barriers and dynamical stability.

At 100 GPa, the previously reported Na−N phases, including *Pmn*2_1_ NaN_5_, *Pbam* Na_2_N_5_, *Cmmm* NaN_2_, *Cmmm* NaN, and *Fm*‐3 *m* Na_3_N, are readily identified in our structural search. The optimized cell parameters of these phases are consistent with previous experimental and theoretical results.[^25^, ^26^] These results demonstrate that our structural prediction method and adopted PBE functional are appropriate for the Na−N system. In addition to reproducing the known Na−N phases, we identified six novel Na−N structures: *C*2/*c* and *C*2/*m* Na_8_N, *P*6_3_/*mmc* Na_5_N, *Immm* Na_2_N, *I*4 1/*amd* NaN_2_, and *Imma* NaN. To provide more information for experimental synthesis, the enthalpy difference analysis was extended to determine the survival pressure range for each known and newly predicted Na−N compound (Figure 1b).

Most of stable phases, such as NaN_5_, Na_2_N_5_, Na_2_N, Na_3_N, and Na_5_N, do not experience structural transitions with pressure. However, NaN_2_ and NaN, both of which adopt the *Cmmm* symmetry, undergo phase transitions to *I*4 1/*amd* and *Imma* phases at 272 and 236 GPa, respectively. For the most Na‐rich compound, Na_8_N, it stabilizes in a *C*2/*c* structure at 185 GPa, then transforms to a *C*2/*m* phase at 274 GPa. Notably, *P*6/*mmm* Na_5_N, isostructural to the previously reported Li_5_N,^[23]^ has a formation enthalpy of 59.7 meV/atom above the convex hull, relative to neighboring reference phases Na_3_N and Na. However, Na_5_N is found to be spontaneous exothermic reaction relative to Na and N, with a corresponding formation enthalpy of −1.288 eV/atom at 100 GPa. This suggests that Na_5_N is thermodynamically favorable compared to its constituent elements. It is important to note that metastable materials, such as Na_5_N, account for approximately 20 % of the synthesized materials in the inorganic crystalline materials. Some of these metastable materials have formation energies as high as 70.0 meV/atom.^[41]^ This indicates that *P*6/*mmm* Na_5_N may still be viable under specific conditions. Finally, all the predicted Na−N phases are dynamically stable, as confirmed by the absence of any imaginary phonon modes throughout the whole Brillouin zone (Figure S3).

### 2.2 Crystal Structures and Phase Transition Mechanism of Na_5_N

**Figure 2:**
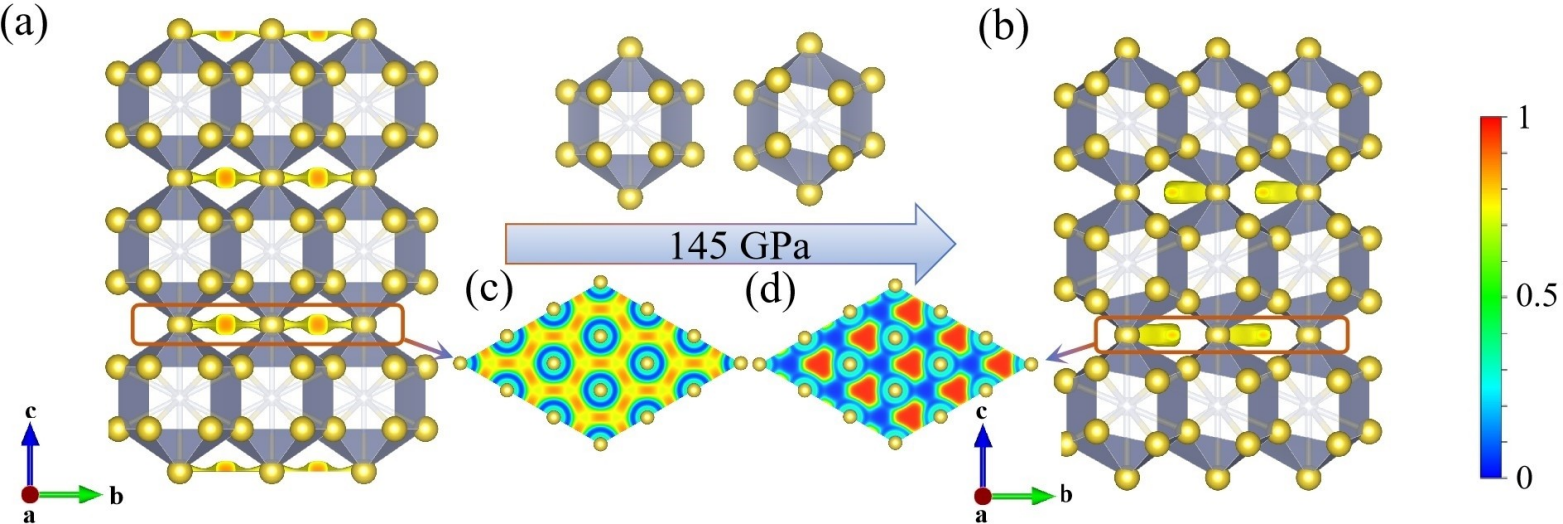
Crystal structures of (a) *P*6/*mmm* Na_5_N at 65 GPa and (b) *P*6_3_/*mmc* Na_5_N at 300 GPa. ELF maps on the (001) plane in (c) *P*6/*mmm* Na_5_N and in (d) *P*6_3_/*mmc* Na_5_N. Yellow and gray spheres represent sodium and nitrogen atoms, respectively.

Herein, we focus on Na_5_N due to the changes in its interstitial anionic electrons (IAEs) configuration caused by structural phase transitions, which provide a good model for analyzing the impact of interstitial electrons on stability. The Na_5_N phase exhibits hexagonal symmetry (space group *P*6/*mmm*, 1 formula unit per cell, Figure 2a), and is isostructural to Li_5_N,^[23]^ in which each N atom is 14‐fold coordinated. This forms a double‐capped Na_14_N hexagonal prism with Na−N distances ranging from 2.27 to 2.40 Å at 65 GPa. Face‐sharing Na_14_N polyhedra arrange into layers in the *ab* plane, and these layers are interconnected by Na atoms sharing vertices along the *c*‐axis. Upon compression, *P*6_3_/*mmc* structure becomes more stable than *P*6/*mmm* above 145 GPa. The Na_14_N hexagonal prism, as a building block, is also found in *P*6_3_/*mmc* Na_5_N (Figure 2b), but the spatial orientation of the Na_14_N has shifted. This change in orientation leads to a reconfiguration of the interstitial electron distribution, as discussed below.

**Figure 3:**
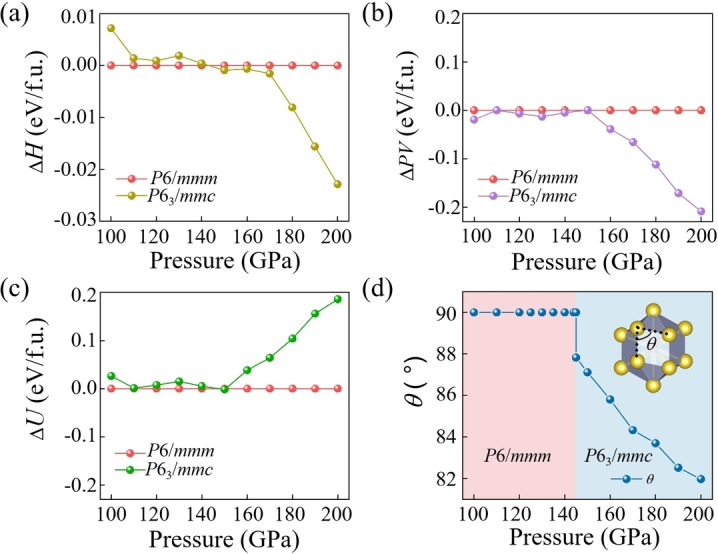
The difference in (a) enthalpy (*H*), (b) pressure‐volume (*PV*), and (c) internal energy (*U*) terms between *P*6_3_/*mmc* and *P*6/*mmm* Na_5_N as a function of pressure from 100 to 200 GPa. (d) The angle (*θ*) of a double‐capped hexagonal prism varies with pressure.

To further investigate the origin of the phase transition, we analyze the enthalpy components, including the internal energy (*U*) and the pressure‐volume (*PV*) term, for the *P*6_3_/*mmc* phase in comparison to the *P*6/*mmm* phase, as shown in Figures 3a–c. The *P*6_3_/*mmc* phase exhibits a more densely packed structure than the *P*6/*mmm* phase, as evidenced by the variation in the pressure dependence of the *PV* term (Figure 3b). This phase transition leads to a reduction in volume by 0.055 % relative to the *P*6*/mmm* structure at 145 GPa, leading to a lower enthalpy. The feature emphasizes the crucial role of volume reduction in stabilizing the structure. Additionally, we investigate the change in the dip‐angle (*θ*) of the Na_14_N polyhedron with pressure. As shown in Figure 3d, the *θ* remains relatively stable between 100 GPa and 145 GPa but decreases significantly above 145 GPa. This rapid change in *θ* further supports the occurrence of a phase transition from the *P*6/*mmm* to the *P*6_3_/*mmc* structure under high pressure.

### 2.3 Interstitial Anionic Electron Topology of Na_5_N

**Figure 4:**
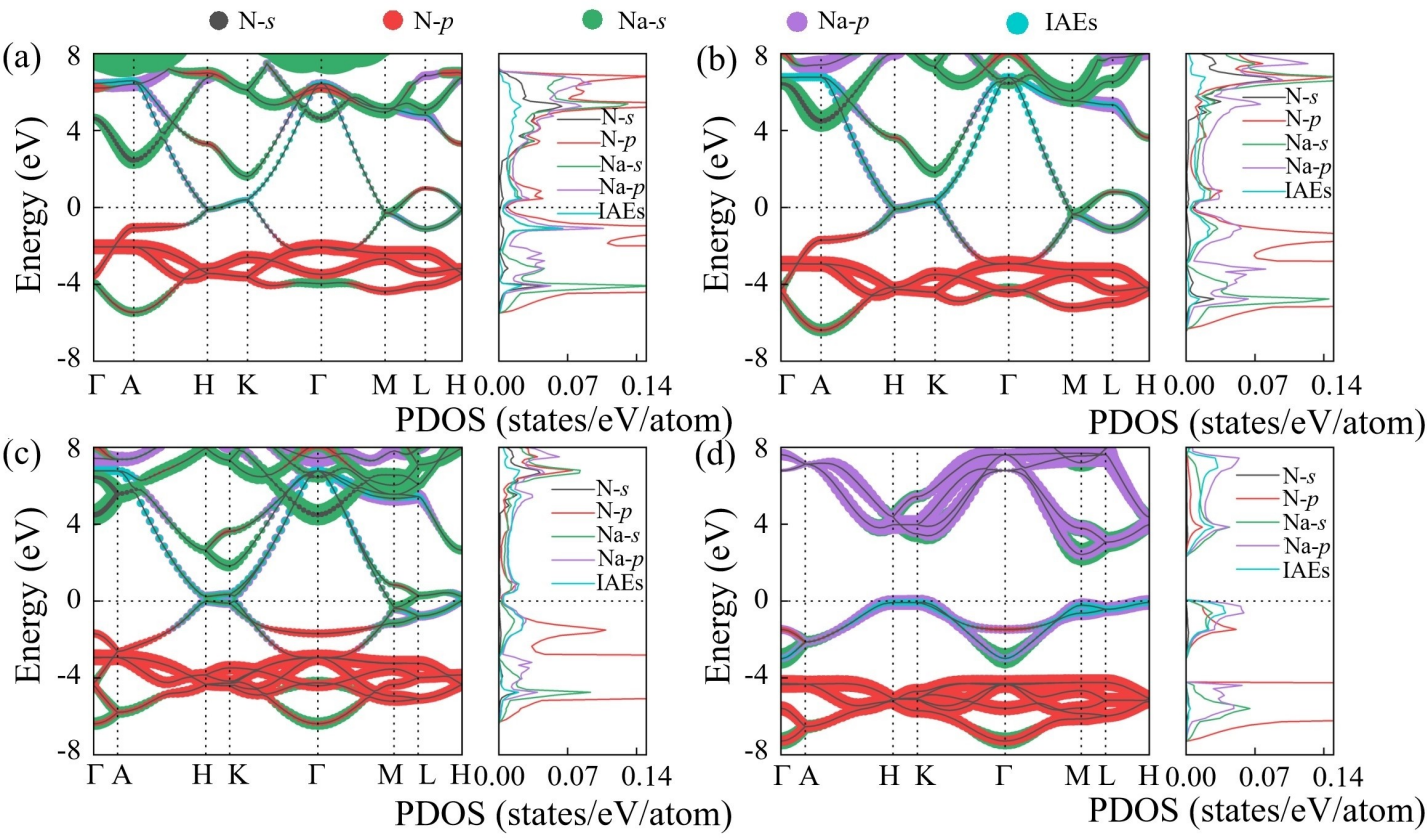
The orbital‐projected electronic band structures and projected density of states (PDOS) of (a)–(b) *P*6/*mmm* Na_5_N at 65 and 145 GPa, (c)–(d) *P*6_3_/*mmc* Na_5_N at 145 and 500 GPa.

It is known that one nitrogen atom can maximally accept up to three electrons from metals, forming a closed shell. However, in Na‐rich Na_5_N, the nitrogen atom cannot accommodate all the electrons donated by sodium, resulting in an excess of electrons within the lattice interstitials, as observed in other electrides like Li_5_N,^[23]^ Li_6_P,^[16]^ and Li_6_C.^[42]^ Interestingly, the two Na‐rich phases of Na_5_N show distinct topology of IAEs located at the interstices between adjacent Na_14_N layers, as confirmed by the electron localization function (ELF) (Figure S4). In the *P*6/*mmm* Na_5_N, the IAEs form a two‐dimensional (2D) graphene‐like configuration (Figure 2c), while in the *P*6_3_/*mmc* Na_5_N, the IAEs adopt a zero‐dimensional (0D) triangle‐like configuration (Figure 2d). The 0D IAEs can be viewed as located in the center of the graphene‐like configuration. The IAE with a 2D graphene‐like configuration is conductive in the plane, which primarily accounts for its metallic nature (Figures 4a–b). The topological transition from 2D to 0D strengthens the interaction between Na and IAEs, leading to a stronger hybridization with Na and IAEs, which results in the emergence of semiconducting behavior (Figures 4c–d). This enhanced hybridization is beneficial for structural stability. Bader charge analysis further elucidates the charge transfer from Na to N and the lattice interstitials. In particular, the amount of anionic charge in the *P*6_3_/*mmc* Na_5_N phase (0.94 e at 300 GPa) is significantly larger than that in the *P*6/*mmm* phase (0.87 e at 65 GPa) (Table. S1). This analysis suggests that the increasing pressure causes a shift from a 2D to a 0D configuration for the IAEs, which increases the interaction between cation and IAEs, leading to a structural distortion that reduces the volume and enhances the structure stability.

### 2.4 Electronic Properties of Na_5_N

Considering that electrides can exhibit elusive electronic conductivity, such as metallic properties in low‐pressure Li,^[9]^ whereas high‐pressure Li is semiconducting,^[43]^ as well as superconductivity, as in Li_6_C,^[42]^ Li_6_P,^[16]^ and Li_6_Al,^[44]^ we subsequently explore the electronic properties of the *P*6/*mmm* and *P*6_3_/*mmc* phases of Na_5_N. The *P*6/*mmm* phase remains metallic in its stable pressure region, as evidenced by several bands crossing the Fermi level, where the Na‐3*p* and N‐2*p* orbits make the main contribution at the Fermi level (Figures 4a–b). Meanwhile, there is pronounced overlap between Na‐3*p* and N‐2*p* below the Fermi level, implying the strong interaction between Na and N atoms. In contrast, the *P*6_3_/*mmc* phase undergoes a metal‐to‐semiconductor transition with pressure (Figures 4c–d). Specifically, *P*6_3_/*mmc* Na_5_N exhibits metallic at 145 GPa, but becomes an indirect‐band‐gap semiconductor above 170 GPa. The orbital‐projected electronic band and PDOS (Figure 4d) show that the 3*p* orbital of Na dominantly contributes to the valence band maximum (VBM), whereas the conduction band minimum (CBM) comes from Na‐3*s* orbital at 500 GPa. This abnormal pressure‐dependent electronic behavior can be explained by the following: as pressure increases, the electrons provided by Na atoms increase, while the electrons received by the N atoms decrease, leading to an accumulation of charge in the interstitial electrons. Therefore, the increase of IAEs further leads to enhanced hybridization between IAEs and Na. Specifically, as pressure increases, the IAEs below the Fermi level become increasingly localized, while the degree of hybridization with Na s/*p*/*d* orbital also increases. Additionally, the band gap of the semiconductor increases with pressure (Figure S6), as observed in FeH_6_ ^[45]^ and GaAs.^[46]^

### 2.5 Structures and Electronic Properties of Na_8_N and Na_2_N

**Figure 5:**
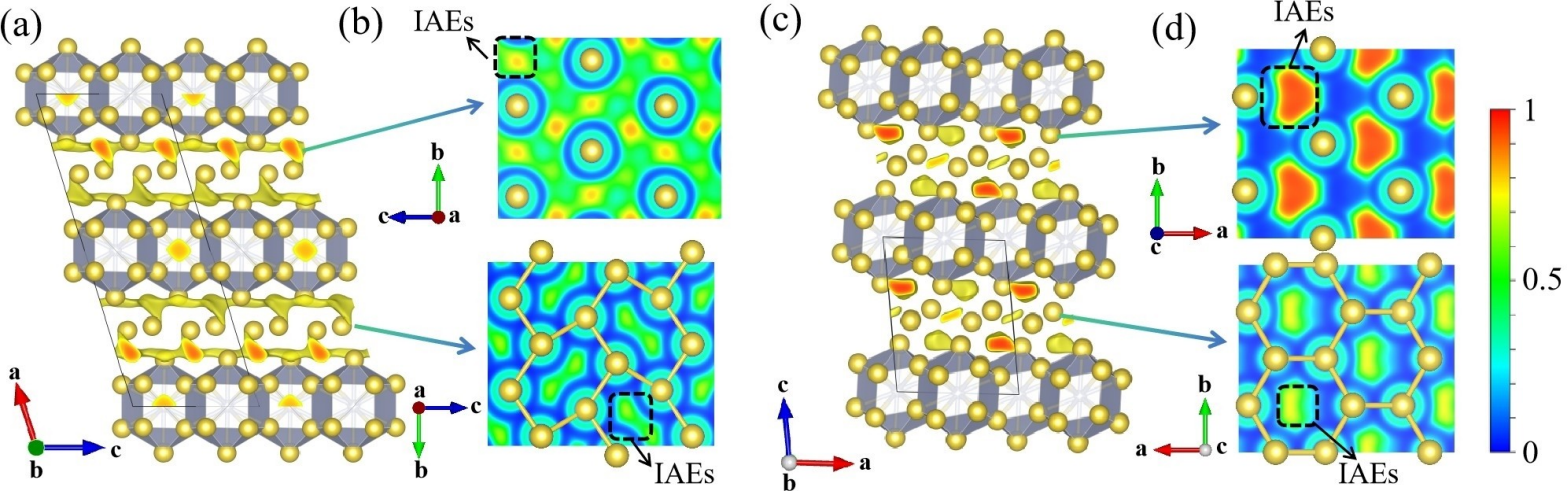
Crystal structures and ELF of *C*2/*c* Na_8_N (a and b) and *C*2/*m* Na_8_N (c and d). Yellow and gray spheres represent sodium and nitrogen atoms, respectively.

The Na‐richest Na_8_N stabilizes two monoclinic phases with *C*2/*c* and *C*2/*m* symmetry, respectively. Both phases share a similar building block of Na_14_N polyhedral, as appeared in Na_5_N, with a face‐sharing arrangement. Compared to Na_5_N, the excess Na atoms in Na_8_N form rectangular‐edge‐sharing layers in the *C*2/*c* phase and corrugated honeycomb layers in the *C*2/*m* phase (Figure 5). As the Na content increases, there are more charge transfers from Na to lattice interstices. Consequently, the excess electrons, amounting to 2.18 e per formula unit (higher than that 0.87 and 0.94 e in Na_5_N), are located in the interstices between adjacent Na_14_N units, forming 0D electrides with a “cloud‐like” shape in the *C*2/*c* phase. Upon structural transition, the shape of the IAEs evolves from a cloud‐like to triangular‐ and ship‐like shapes. Interestingly, *C*2/*m* shows the highest concentration of IAEs among the Na−N compounds (i. e., 2.43 e at 300 GPa). The *C*2/*c* phase is metallic, while the *C*2/*m* phase exhibits weak superconductivity, yielding a *T*_c_ value of 0.23 K with an electron‐phonon coupling (EPC) parameter of 0.33 at 300 GPa, calculated using the Allen‐Dynes modified McMillan formula^[47]^ with a Coulomb pseudopotential of *μ* ^*^=0.1. The present *T*_c_ value is comparable to 0.43 K for Li_7_Te,^[48]^ 1.1 K for Li_5_Si,^[49]^ and 3.4 K for Ba_2_N.^[50]^ Notably, applying strain can increase the *T*_c_ of Ba_2_N to 10.8 K,^[50]^ suggesting that the superconducting transition temperature of *C*2/*m* Na_8_N may also be enhanced under similar conditions. The increased net charge of IAEs combines with the Na electrons to form a hybrid conductive network, which is also clearly seen in the PDOS (Figure S7b). This further strengthens their coupling to the vibrations of the surrounding Na atoms, as evidenced by obvious Na‐dominated low‐frequency phonon softening (Figure S10a). Eliashberg spectral function and phonon density of states (PHDOS) show that Na‐dominated low frequency phonons (0–13.2 THz) make the main contribution of ∼91.5 % (Figure S10b). As a result, the electronic properties among the two Na_8_N phase can be manipulated by adjusting the IAEs charge. In *Immm* Na_2_N, each Na atom is coordinated with six N atoms, and the neighboring Na atoms form a one‐dimensional chain along the *c*‐axis, with a Na−Na distance of 1.971 Å at 200 GPa (Figures S11a–S11b). Moreover, further electronic properties indicate that *Immm* Na_2_N is a semiconductor with an indirect band gap of 3.5 eV at 200 GPa (Figure S11c).

## 3 Conclusions

In summary, our comprehensive study of the Na−N system under high pressure reveals a rich diversity of phase transitions and electronic behaviors. We identify several novel Na−N phases, including Na_8_N, Na_5_N, and Na_2_N, confirming their structural stability and potential for experimental synthesis. Notably, Na_5_N undergoes significant changes in its interstitial electron configuration with pressure, resulting in a shift from a 2D to a 0D electron topology, which enhances structural stability. The phase transition from *P*6/*mmm* to *P*6_3_/*mmc* in Na_5_N is driven by volume reduction and reconfiguration of Na_14_N polyhedral. *P*6_3_/*mmc* experiences a metal‐to‐semiconductor transition with pressure. In *C*2/*m* Na_8_N, excess Na atoms form honeycomb layers, and the interstitial electrons adopt triangular‐ and ship‐like configurations. Notably, *C*2/*m* Na_8_N exhibits superconductivity with a *T_c_* of 0.23 K at 300 GPa, which may be influenced by the coupling of hybridized electrons of IAEs and Na with Na‐derived low‐frequency phonons. More broadly, the extent to which IAEs contribute to superconductivity remains an interesting question for further investigation. Additionally, *Immm* Na_2_N demonstrates semiconductor behavior with a large indirect band gap. These findings provide crucial insights into the high‐pressure chemistry of Na−N compounds and their potential applications.

## Conflict of Interests

The authors declare no conflict of interest.

4

## Supporting information

As a service to our authors and readers, this journal provides supporting information supplied by the authors. Such materials are peer reviewed and may be re‐organized for online delivery, but are not copy‐edited or typeset. Technical support issues arising from supporting information (other than missing files) should be addressed to the authors.

Supporting Information

## Data Availability

The data that support the findings of this study are available in the supplementary material of this article.
