# Supplementary material for: High‐Pressure Stability and Electronic Properties of Sodium‐Rich Nitrides: Insights from First‐Principles Calculations
Source: Chemphyschem. 2025 Mar 6;26(10):e202401150. doi: 10.1002/cphc.202401150 (PMC12091849; doi:10.1002/cphc.202401150)
Supplement: Supplementary file 1 — Supporting Information [file CPHC-26-e202401150-s001.pdf]

ChemPhysChem

Supporting Information

High-Pressure Stability and Electronic Properties of Sodium-Rich Nitrides: Insights from First-Principles Calculations

Qiuyue Li, Qiuping Yang, Shuai Han, Fei Li,* Yansun Yao,* and Guochun Yang

Supplemental Material

High-Pressure Stability and Electronic Properties of Sodium-rich Nitrides: Insights from First-Principles Calculations

Qiuyue Li+,[a], Qiuping Yang+,[b], Shuai Han[a], Fei Li*[a], Yansun Yao*[c], Guochun Yang[a]

[a] Q. Li, S. Han, Dr. F. Li*, Prof. Dr. G. Yang
State Key Laboratory of Metastable Materials Science & Technology and Hebei Key Laboratory of Microstructural Material Physics, School of Science, Yanshan University, Qinhuangdao 066004, China
Email: lifei718@ysu.edu.cn

[b] Q. Yang
Key Laboratory of Materials Modification by Laser, Ion and Electron Beams (Dalian University of Technology), Ministry of Education, Dalian 116024, China

[c] Prof. Dr. Y. Yao*
Department of Physics and Engineering Physics, University of Saskatchewan, Saskatoon, Saskatchewan, Canada S7N 5E2
E-mail: yansun.yao@usask.ca

[+] These authors contributed equally.

Index··Page

1. Computational Details···3

2. Calculated Birch-Murnaghan equation of states for *P*6/*mmm* NaN_3································6

3. Structural Description···7

4. Phonon dispersion curves of Na-N compounds···8

5. Crystal structures and ELF of *P*6/*mmm* Na_5N and $P6_3/mmc$ Na_5N·······························9

6. Phonon dispersion curves for *P*6/*mmm* Na_5N and $P6_3/mmc$ Na_5N at different pressures·······9

7. The band-gap evolution of $P6_3/mmc$ Na_5N with pressure··10

8. Energy bands and DOS of *C*2/*c* Na_8N at 200 and 300 GPa··10

9. Phonon dispersion curves for *C*2/*c* Na_8N at 200 GPa and *C*2/*m* Na_8N at 300 GPa··············10

10. The difference in enthalpy (*H*), pressure-volume (*PV*) term, and internal energy (*U*) between *C*2/*m* Na_8N and *C*2/*c* Na_8N··11

11. Phonon linewidth, PHDOS, $\alpha^2F(\omega)$, and frequency-dependent EPC parameter $\lambda(\omega)$ of *C*2/*m* Na_8N at 300 GPa··11

12. Crystal structures, ELF, electronic band structure and PDOS of *Immm* Na_2N at 200 GPa·····11

13. Bader charges of Na_5N, Li_5N, and Na_8N··12

14. Structural parameters of the predicted stable Na-N compounds························12

Computational Details

Our structural prediction approach is based on a global minimization of free energy surfaces merging *ab initio* total-energy calculations with CALYPSO (Crystal structure AnaLYsis by Particle Swarm Optimization) methodology as implemented in the CALYPSO code. The structures of stoichiometry Na_xN_y (x = 1-8, y = 1-6) were searched with simulation cell sizes of 1-4 formula units (f.u.) at at 0 K and the considered pressures of 100, 200 and 300 GPa. In the first step, random structures with certain symmetry are constructed in which atomic coordinates are generated by the crystallographic symmetry operations. Local optimizations using the VASP code were done with the conjugate gradients method and stopped when Gibbs free energy changes became smaller than 1×10^{-5} eV per cell. After processing the first generation structures, 60% of them with lower Gibbs free energies are selected to construct the next generation structures by PSO (Particle Swarm Optimization). 40% of the structures in the new generation are randomly generated. A structure fingerprinting technique of bond characterization matrix is applied to the generated structures, so that identical structures are strictly forbidden. These procedures significantly enhance the diversity of the structures, which is crucial for structural global search efficiency. In most cases, structural searching simulations for each calculation were stopped after generating 1000 ~ 1200 structures (e.g., about 20 ~ 30 generations).

To further analyze the structures with higher accuracy, we select a number of structures with lower enthalpies and perform structural optimization using density functional theory within the generalized gradient approximation as implemented in the VASP code. The cut-off energy for the expansion of wavefunctions into plane waves is set to 750 eV in all calculations, and the Monkhorst-Pack k-mesh with a maximum spacing of $2\pi \times 0.03$ Å^{-1} was individually adjusted in reciprocal space with respect to the size of each computational cell. This usually gives total energies well converged within ~1 meV/atom. The electron-ion interaction was described by using all-electron projector augmented-wave method (PAW) with $2p^6 3s^1$ valence electrons

for Na atom, $2s^22p^3$ valence electrons for N atom, respectively. The formation enthalpy (ΔH), relative to the elemental solids (Na and N), was calculated at each considered pressure according to the equation below:

$$\Delta H(Na_xN_y) = [H(Na_xN_y) - xH(Na) - yH(N)]/(x + y),$$

where $H = U + PV$ is the enthalpy of each composition, and ΔH is the formation enthalpy per atom of the given compound. Here, U, P, and V are the internal energy, pressure, and volume, respectively.

In order to further test the reliability of the adopted pseudopotentials for Na and N, the validity of the projector augmented wave (PAW) pseudopotentials from the VASP library is checked by comparing the calculated Birch-Murnaghan equation of state with that obtained from the full-potential linearized augmented plane-wave method (LAPW) using local orbitals (as implemented in WIEN2k). The Birch-Murnaghan equation of states derived from PAW and LAPW methods are almost identical (Figure. S1). Thus, our adopted pseudopotentials are feasible in the range of 0~200 GPa.

The phonon spectra and electron-phonon coupling calculations are carried out with the density functional perturbation (linear response) theory as implemented in the QUANTUM ESPRESSO package. We employ the ultrasoft pseudopotentials with $2s^22p^63s^1$, and $2s^22p^3$ as valence electrons for Na, and N atoms, respectively. The kinetic energy cutoff for wave-function expansion is chosen as 80 Ry. To reliably calculate electron-phonon coupling in metallic systems, we need to sample dense k-meshes for the electronic Brillouin zone integration and enough q-points for evaluating the average contributions from the phonon modes. Here, we used a 12 × 12 × 4 k-mesh and 6 × 6 × 2 q-mesh for calculating the superconducting T_c of 0.23 K. We have calculated the superconducting T_c as estimated from the McMillan-Allen-Dynes formula

$$T_C = \frac{\omega_{\log}}{1.2k_B}\exp\left[-\frac{1.04(1+\lambda)}{\lambda-\mu^*(1+0.62\lambda)}\right].$$

Here, k_B is the Boltzmann constant and μ^* is the Coulomb pseudopotential ($\mu^* = 0.1$). The electron-phonon coupling constant, λ, and the logarithmic average phonon

frequency, $\omega_{\log}$, are calculated by the Eliashberg spectral function for electron-phonon interaction:

$$\alpha^2 F(\omega) = \frac{1}{N(E_F)} \sum_{kq,v} \left| g_{k,k+q,v} \right|^2 \delta(\varepsilon_k)\delta(\varepsilon_{k+q})\delta(\omega - \omega_{q,v})$$

where $\lambda = 2\int d\omega \frac{\alpha^2 F(\omega)}{\omega}$; $\omega_{\log} = \exp\left[\frac{2}{\lambda}\int \frac{d\omega}{\omega}\alpha^2 F(\omega)\ln(\omega)\right]$. Herein, $N(E_F)$ is the electronic density of states at the Fermi level, $\omega_{q,\,v}$ is the phonon frequency of mode v and wave vector q, and $|g_{k,k+q,v}|$ is the electron-phonon matrix element between two electronic states with momenta k and $k + q$ at the Fermi level.

Supporting Figures

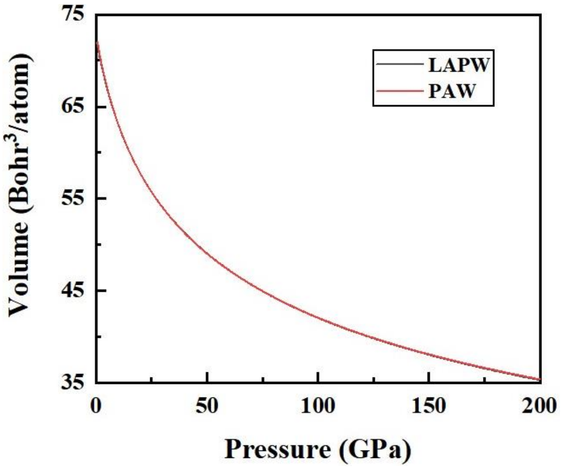

Fig. S1. Comparison of the fitted Birch-Murnaghan equation of states for *P*6/*mmm* NaN_3 using calculated results with the PAW pseudopotentials and full-potential LAPW methods.

Structural Description

In addition to the Na-N compounds reported in manuscript, here, we describe the structures of other disclosed Na-N phases. In *Imma* NaN, N atoms form zigzag chains along the *a*-axis, with the N-N bond length of 1.35 Å at 300 GPa, and Na atoms are distributed between adjacent N chains. Whereas for *I*4_1/*amd* NaN_2, N atoms form zigzag N chains along the *a*-axis and *b*-axis directions with N-N distance of 1.27 Å at 300 GPa .

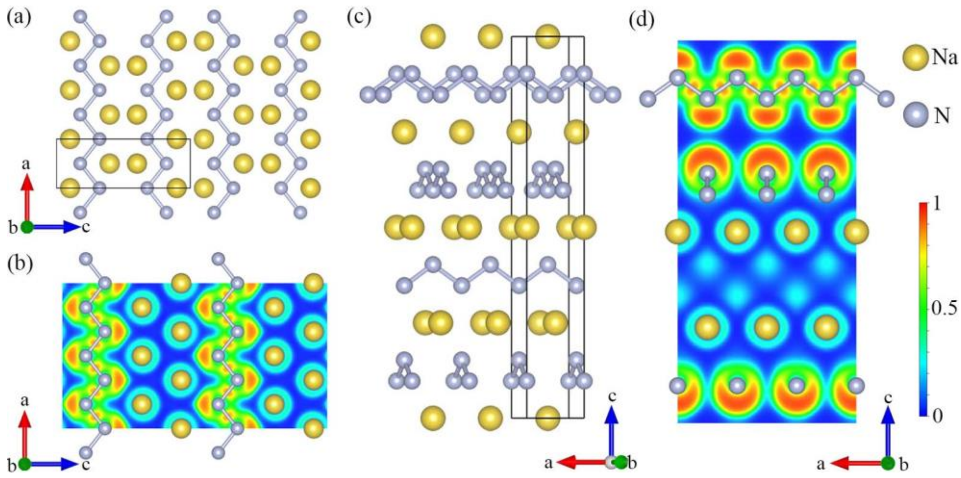

Fig. S2. Crystal structures of (a) *Imma* NaN at 300 GPa and (c) *I*4_1/*amd* NaN_2 at 300 GPa. The ELF of (b) *Imma* NaN at 300 GPa and (d) *I*4_1/*amd* NaN_2 at 300 GPa.

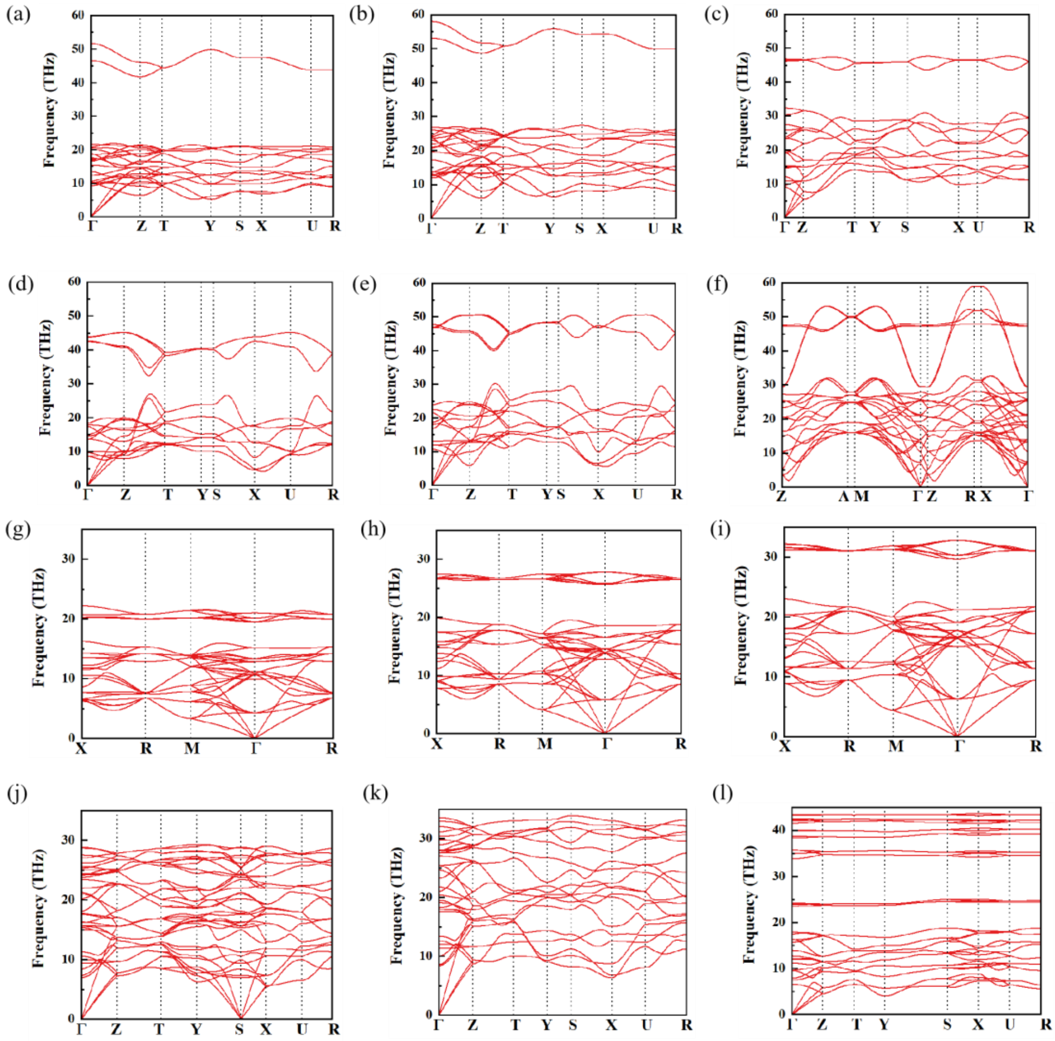

Fig. S3. Phonon dispersion curves for (a) *Cmmm* NaN at 100 GPa, (b) *Cmmm* NaN at 200 GPa, (c) *Imma* NaN at 300 GPa, (d) *Cmmm* NaN_2 at 100 GPa, (e) *Cmmm* NaN_2 at 200 GPa, (f) *I*4_1/*amd* NaN_2 at 300 GPa, (g)-(i) *Fm*-3*m* Na_3N at 100-300 GPa, (j)-(k) *Immm* Na_2N at 200-300 GPa, and (l) *Pmn*2_1 NaN_5 at 100 GPa.

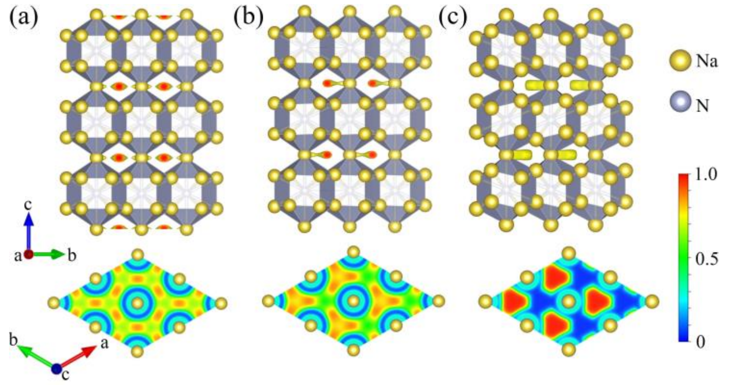

Fig. S4. Crystal structures and ELF maps on the (001) plane of (a) *P*6/*mmm* Na_5N at 145 GPa, (b) *P*6$_3$/*mmc* Na_5N at 145 GPa, and (c) *P*6$_3$/*mmc* Na_5N at 500 GPa. Yellow and gray spheres represent sodium and nitrogen atoms, respectively.

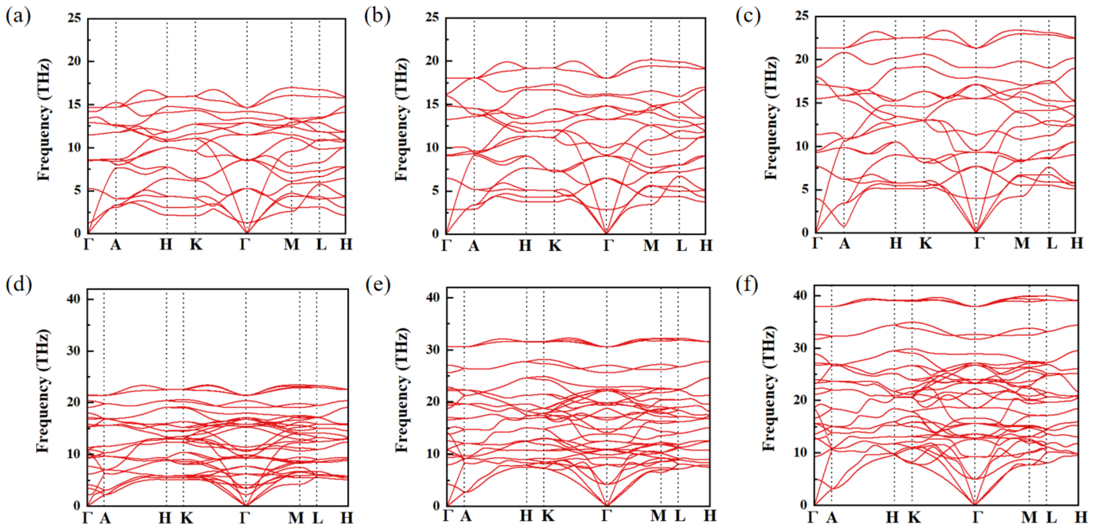

Fig. S5. Phonon dispersion curves for *P*6/*mmm* Na_5N at (a) 65, (b) 100, and (c) 145 GPa. Phonon dispersion curves for *P*6$_3$/*mmc* Na_5N at 145, 300, and 500 GPa.

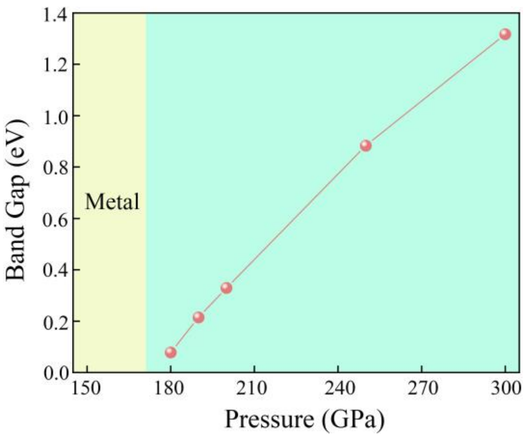

Fig. S6. Pressure-dependent electronic properties of $P6_3/mmc$ Na_5N.

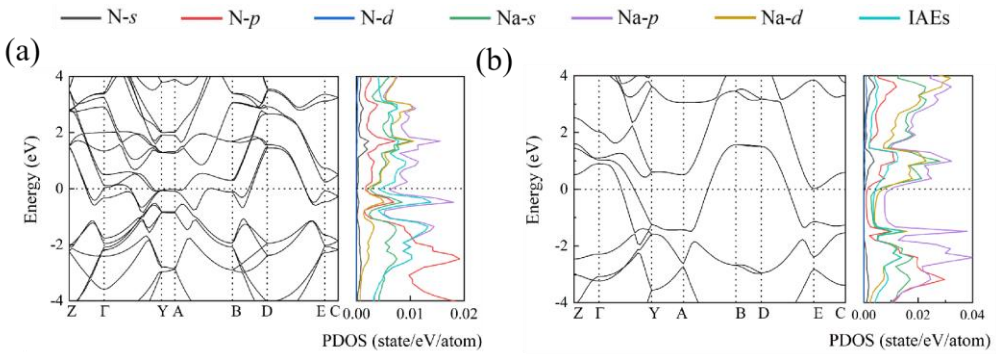

Fig. S7. Electronic band structure and PDOS of (a) $C2/c$ Na_8N at 200 GPa and (b) $C2/m$ Na_8N at 300 GPa.

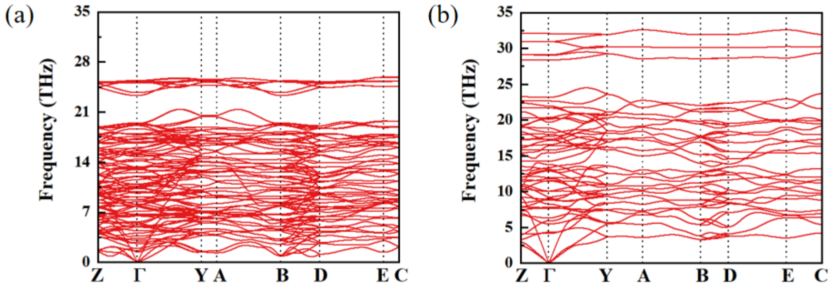

Fig. S8. Phonon dispersion curves for (a) $C2/c$ Na_8N at 200 GPa and (b) $C2/m$ Na_8N at 300 GPa.

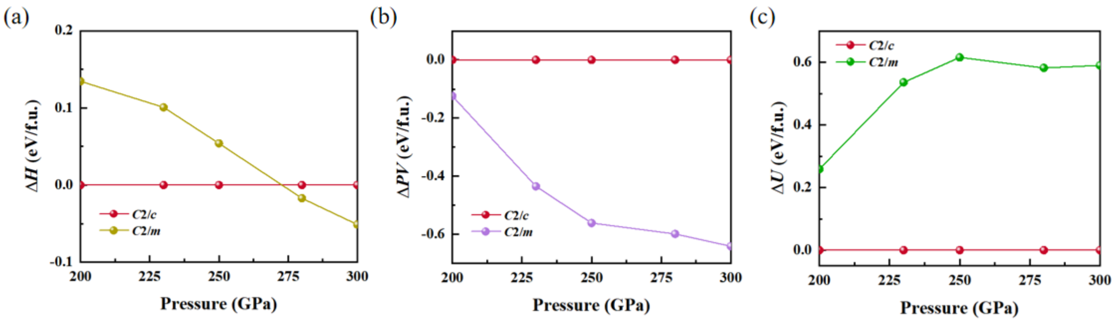

Fig. S9. The difference in (a) enthalpy (*H*), (b) pressure-volume (*PV*) term, and (c) internal energy (*U*) between *C*2/*m* Na_8N and *C*2/*c* Na_8N as a function of pressure from 200 to 300 GPa.

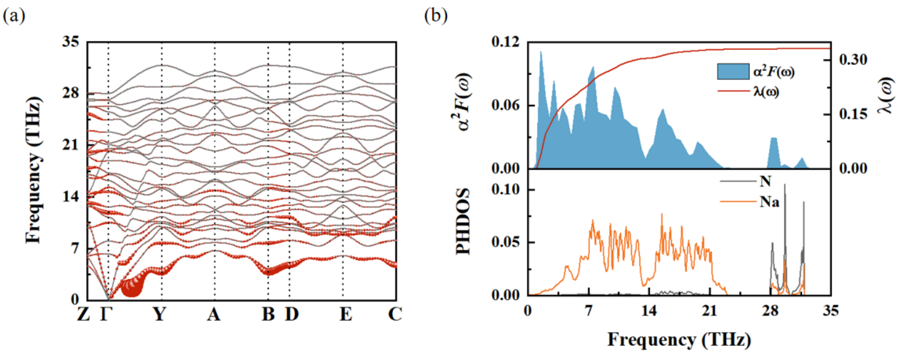

Fig. S10. (a) Phonon linewidth, (b) phonon density of states (PHDOS), Eliashberg spectral function $\alpha^2F(\omega)$, and frequency-dependent electron-phonon coupling (EPC) parameter $\lambda(\omega)$ of *C*2/*m* Na_8N at 300 GPa.

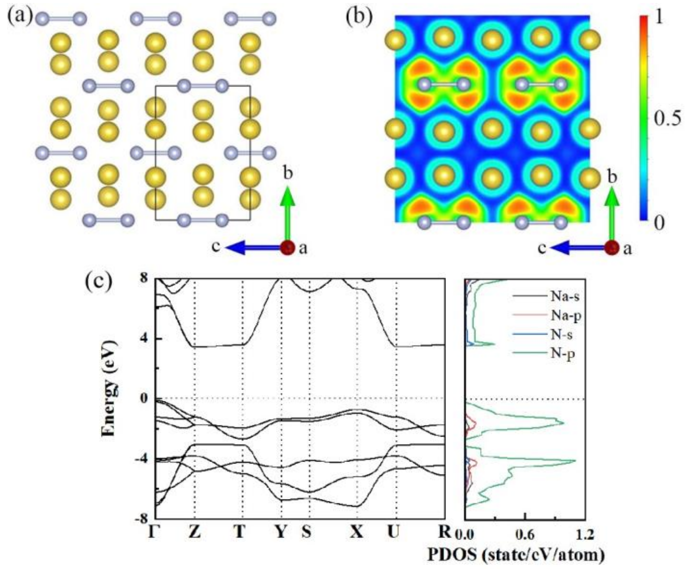

Fig. S11. (a) Crystal structures, (b) ELF maps on the (001) plane, (c) electronic band structure and projected density of states (PDOS) of *Immm* Na_2N at 200 GPa.

Supporting Tables

Table S1. Bader charges of Na_5N and Na_8N.

Phases	Pressure (GPa)	Atoms	Charge (e/f.u.)	Δe
*P*6/*mmm* Na_5N	65	Na	31.92	−3.08
		N	7.21	2.21
		IAEs	0.87	0.87
	145	Na	31.97	−3.03
		N	7.20	2.20
		IAEs	0.83	0.83
P6_3/*mmc* Na_5N	145	Na	32.01	−2.99
		N	7.19	2.19
		IAEs	0.80	0.80
	300	Na	31.89	−3.11
		N	7.17	2.17
		IAEs	0.94	0.94
	500	Na	31.86	−3.14
		N	7.15	2.15
		IAEs	0.99	0.99
*C*2/*c* Na_5N	200	Na	51.64	−4.36
		N	7.18	2.18
		IAEs	2.18	2.18
C2/*m* Na_5N	300	Na	51.41	−4.59
		N	7.16	2.16
		IAEs	2.43	2.43

Table S2. Structural parameters of the predicted stable Na-N compounds.

Phase	Pressure (GPa)	Lattice parameters (Å, °)	atoms	Atomic coordinates *x*	*y*	*z*
*I*4_1/*amd*	300	$a = b = 2.0674$	Na(4b)	0.0000	1.0000	0.5000
NaN_2		$c = 13.2814$	N(8e)	−0.5000	1.0000	0.6526
		$\alpha = \beta = \gamma = 90°$				
Imma	300	$a = 2.1280$	Na(4e)	0.00000	0.2500	0.1019
NaN		$b = 3.2070$	N(4e)	0.00000	−0.2500	0.3221
		$c = 5.8177$				
		$\alpha = \beta = \gamma = 90°$				
Immm	200	$a = 3.2193$	Na(4h)	0.0000	0.3421	0.5000

Na_2N		*b* = 5.5951	Na(4g)	0.5000	0.8206	0.5000
		c = 3.9352	N(4i)	0.0000	0.0000	0.7000
		α = *β* = *γ* = 90°				
*P*6/*mmm*	100	*a* = *b* = 3.5077	Na(4h)	0.3333	0.6667	0.7130
Na_5N		*c* = 4.8183	Na(4g)	0.0000	0.0000	0.0000
		α = *β* = 90°	N(4i)	0.0000	0.0000	0.5000
		γ = 120°				
$P6_3/mmc$	300	*a* = *b* = 3.1228	Na(4h)	0.6667	0.3333	0.8680
Na_5N		*c* = 8.4728	Na(4h)	0.3333	0.6667	0.9113
		α = *β* = 90°	Na(4g)	0.0000	0.0000	0.7500
		γ = 120°	N(4i)	0.0000	0.0000	0.5000
*C*2/*c*	200	*a* = 14.6755	Na(8f)	0.14896	0.2474	1.3685
Na_8N		*b* = 3.2987	Na(8f)	0.75665	0.3962	1.3538
		c = 5.6751	Na(8f)	0.93191	0.2506	0.5307
		α = *γ* = 90°	Na(8f)	0.07162	0.7520	1.1381
		β = 107.26°	N(4e)	0.00000	0.7492	0.7500
*C*2/*m*	300	*a* = 5.2888	Na(4i)	0.16157	0.0000	0.4608
Na_8N		*b* = 3.1952	Na(4i)	0.19890	0.0000	0.1712
		c = 6.7159	Na(4i)	0.14701	0.0000	0.8863
		α = *γ* = 90°	Na(4i)	0.44738	0.0000	0.6959
		β = 96.58°	N(2b)	0.00000	0.5000	0.0000
